# Expression levels of circulatory mir-185-5p, vascular endothelial growth factor, and platelet-derived growth factor target genes in endometriosis

**DOI:** 10.18502/ijrm.v13i5.7155

**Published:** 2020-05-31

**Authors:** Mohammad Hossein Razi, Maryam Eftekhar, Nasrin Ghasemi, Mohammad Hasan Sheikhha, Ali Dehghani Firoozabadi

**Affiliations:** ^1^Research and Clinical Center for Infertility, Yazd Reproductive Sciences Institute, Shahid Sadoughi University of Medical Sciences, Yazd, Iran.; ^2^Biotechnology Research Center, International Campus, Shahid Sadoughi University of Medical Sciences, Yazd, Iran.; ^3^Abortion Research Center, Yazd Reproductive Sciences Institute, Shahid Sadoughi University of Medical Sciences, Yazd, Iran.; ^4^Yazd Cardiovascular Research Center, Shahid Sadoughi University of Medical Sciences, Yazd, Iran.

**Keywords:** Biomarker, miRNA, Diagnosis, Endometriosis, Angiogenesis.

## Abstract

**Background:**

Using blood-based biomarkers such as microRNAs (miRNAs) may allow particularly effective and minimally invasive diagnosis and treatment of endometriosis. **Objective: **We evaluated the differential expression of circulating miRNA-185-5p (miR-185-5p), vascular endothelial growth factor *(VEGF)*, and platelet-derived growth factor *(PDGF)* target genes between endometriosis and healthy women.

**Materials and Methods:**

25 women with a history of endometriosis (grad III-IV) diagnosed by laparoscopy as the case group and 25 women without endometriosis underwent laparoscopy for ovarian cysts or pelvic pain as the control group were enrolled in this case-control study. Blood samples were obtained, and total RNA was used for high-throughput small RNA sequencing, and this was confirmed by means of quantitative real-time polymerase chain reaction (qRT-PCR).

**Results:**

miRNA expression profiling using non-coding RNA sequencing revealed that one miRNA including miR-185-5p was significantly down-regulated in the case group compared with the controls. The qRT-PCR results showed significant downregulation of the expression level of miR-185-5p (*p* < 0.01) in the plasma of the case group. Receiver operating characteristic (ROC) curve analysis showed the area of miR-185-5p under the ROC curve for endometriosis diagnosis was 0.919 (*p* < 0.001). The RT-PCR results demonstrated that there was no significant difference in the expression of *VEGF* and *PDGF* mRNA of blood samples in the cases compared to the control group (*PDGF*, *p* = 0.09 and *VEGF*, *p* = 0.36).

**Conclusion:**

The low expression of miR-185-5p in the plasma of women with endometriosis could be employed as an important non-invasive biomarker for early detection and screening of endometriosis by blood samples.

## 1. Introduction

The human endometrium is an angiogenic tissue with a large number of endometrial stem cells and notable remodeling potential and is a major source of angiogenesis activators, which are produced by endometrial tissue (1). The important role of angiogenesis and angiogenic factors in the pathophysiology of endometriosis has been confirmed. Angiogenesis is an essential process in the growth, persistence, and metastases of solid tumors (2, 3). Angiogenesis is balanced by proangiogenic and antiangiogenic mediators and is controlled by angiogenesis-related factors, including vascular endothelial growth factor *(VEGF)*, platelet-derived growth factor *(PDGF)*, fibroblast growth factor *(FGF)*, tumor necrosis factor-alpha, transforming growth factor-beta, and the angiopoietins (4, 5). Many types of cells, including fibroblasts, endothelial cells, platelets, inflammatory cells, smooth muscle cells, and tumor cells, produce and release these growth factors (6). The most potent factor for induction of angiogenesis is hypoxia, which induces the expression of *VEGF*. The *VEGF* family in humans consists of five members. Among them, *VEGF-A* and *VEGF-C* are expressed by the endometrium and *VEGF* receptor tyrosine kinases, *VEGFRs* 1 (Flt-1), *VEGFR-2* (KDR, Flk-1), and *VEGFR-3* (Flt-4), which are exclusively expressed in endothelial cells, hematopoietic stem cells, and tumor cells (7, 8).

In addition to *VEGF*, *PDGF* has a critical role in angiogenesis and could be essential during vascularization. Although the fundamental molecular mechanisms of *PDGF* in angiogenesis have not yet been precisely defined, *PDGF* has attracted much attention from researchers from diverse backgrounds. *PDGF* and its specific receptors are expressed by natural and tumor cells and have been considered as an anti-angiogenic target for the treatment of solid tumors (9). The *PDGF* receptor tyrosine kinases, which are known as the α and β subunits, disrupt *PDGF-B/β* signaling and are associated with significant vascular abnormalities in physiologic and pathologic angiogenesis (10, 11).

Recently, it has been said that microRNAs (miRNAs) are commonly dysregulated in endometriosis. Emerging data show that abnormal miRNA expression in relation to endometriosis may facilitate the development and progression of endometriosis by controlling the proliferation, apoptosis, migration, invasion, and estradiol signal transduction in endometriotic cells (3-5).

Little is known about the pathophysiological significance of miRNA-185 (miR-185) in endometriosis. To investigate the molecular mechanisms by which miR-185 may participate in endometriosis, its target genes were searched. Among them, it was found that there was a highly conserved miR-185 responsive element in the 3'-untranslated region (3'-UTR) of *VEGF-A* and *PDGF*. It has been reported that *VEGF-A* is an important angiogenesis regulator that is highly expressed in tumor tissues such as endometrial tissue, and it is proposed that it is closely associated with aggressive tumor features (12, 13).

We speculate that *VEGFA* may be the target gene of miR-185. Considering the importance and the role of miRNAs in pathogenesis as well as the diagnosis and progression of various diseases, and the importance of angiogenesis in the progression of endometriosis, the present study evaluated the differential expression of circulating miR-185-5p and predicted the downstream *VEGF* and *PDGF* target genes between endometriosis women and healthy controls using real-time quantitative reverse transcription polymerase chain reaction (RT-PCR) to provide evidence of the value of miR-185-5p as a new diagnostic biomarker for endometriosis.

In this study, the expression pattern of miR-185 and its target genes *VEGF-A* and *PDGF* were evaluated and compared in whole blood from women with and without endometriosis.

## 2. Materials and Methods

### Participants

25 women aged 18-44 yr old with a history of endometriosis (grade III-IV) diagnosed by laparoscopy as the case group and 25 women in the same age range without endometriosis who underwent laparoscopy for ovarian cysts or pelvic pain, referred to Yazd Khatam-ol-Anbia Clinic of Shahid Sadoughi University of Medical Science, Yazd, Iran, as the control group were enrolled in this cross-sectional study from March 2018 to March 2019. The diagnosis of endometriosis was confirmed histologically, and the severity of endometriosis was classified based on the American Society for Reproductive Medicine (ASRM; 1997) revised classification system (14). In all women with endometriosis, mRNA and miRNA expression of whole blood (WB) and sera were evaluated by real-time quantitative RT-PCR.

### Blood, plasma, and sample preparation and RNA extraction

10 ml venous blood samples were collected in five ethylenediaminetetraacetic acid tubes. Then, one tube was diluted (1:1) with sterile phosphate-buffered saline. Next, standard Ficoll-Hypaque density gradient centrifugation was performed to isolate PBMCs, and plasma was saved for further analysis (15). Two tubes were centrifuged for 15 min at 4000 rpm, and the isolated plasma was saved. Total RNA was isolated from whole blood using the QIAamp RNA Blood Mini Kit (QIAGEN, Germany) with the addition of an on-column DNase I digestion to remove any possible remnants of genomic DNA according to the manufacturer's instructions (16, 17). In addition, total miRNA was isolated from the plasma using the QIAamp miRNA Plasma/Serum Mini kit (QIAGEN, Germany) according to the manufacturer's protocol. The quantity and concentration of extracted RNA were determined using the NanoDrop ND-1000 spectrophotometer (NanoDrop Technologies, Wilmington, DE, USA). The quality of RNA samples was also assessed by 1.5% agarose gel electrophoresis.

### miRNA sequencing

The RNA samples were sent to the Dr. Faghihi's Medical Genetic Center (Shiraz, Iran) for whole-genome miRNA sequencing. The Persian Bayan Gene conducted the miRNA profiling using an Illumina HiSeq sequencer. Illumina TruSeq Small RNA sample prep kits (Illumina, USA) were used to prepare miRNA libraries.

### Reverse transcription and expression analysis

RevertAid Reverse Transcriptase (ThermoFisher Scientific) was used to synthesize single-stranded complementary DNA (cDNA) 1 µg of total RNA following the manufacturer's instructions (18). The cDNA synthesis for microRNA was performed using the polyadenylation method. In this method, poly-A polymerase was applied to the extracted RNA to be polyadenylated and then this RNA used as a template to synthesize the first-strand cDNA. These cDNAs were then diluted to a final concentration of 400 ng/µl for using in the quantitative real-time RT-PCR (qRT-PCR) reactions.

### miRNA sequencing data analysis

For bioinformatics analysis, miRBase was used as a reference library (http://www.mirbase.org/ ftp://mirbase.org/pub/mirbase/CURRENT/README). Low-quality reads were filtered out, while the qualified reads were strictly drawn to all known mature and precursor sequences, in addition to the human genomes. The bioinformatics software was used to analyze the differentially expressed miRNAs between the endometriosis and control subjects. The miRNAs with a *p*-value < 0.05 were considered significantly differential.

### Real-time RT-PCR analysis

StepOnePlus Real-Time PCR system (Applied Biosystems, USA) was used to perform Real-time RT-PCRs in duplicate for the target mRNAs and miR-185. The mean Ct values for the mRNA targets were determined and normalized to glyceraldehyde 3-phosphate dehydrogenase *(GAPDH)* as endogenous controls while for miR-185, small nuclear RNA (SNORD47) was the endogenouse control. The qPCR primer sequences are listed in table I. The relative quantification was performed by 2(-ΔC${}_{T}$): the expression of target genes/housekeeping gene = (1 + E) - C${}_{T}$ target gene / (1 + E) - C${}_{T}$ housekeeping gene. The specificity of the PCR reactions was verified by the generation of a melting curve analysis followed by gel electrophoresis, visualized by SafeView staining.

### Standard curve and efficiency of PCR

The qPCR efficiencies for each of the primer pairs were derived from standard curves, which were obtained from ten-fold serial dilutions of a positive PCR product for *VEGF-A* and *GAPDH* by a usual qRT-PCR. Logarithms of concentrations for each dilution on the *x* axis were plotted against the target gene-cycling threshold (Ct) of serial dilution on the *y* axis. The *VEGF-A* and *GAPDH* efficiencies were 91% and 95.9%, respectively.

**Table 1:** Primer sequences for target and housekeeping genes


**Gene and miRNA name**	**NCBI ID**	**Primer sequence 5'→3'**	**Product length**	**Cycle**	**Ref**
**VEGF-A**	NM_001287044.1	F: AAATGCTTTCTCCGCTCTGA R: CCCACTGAGGAGTCCAACAT	173 bp	40	Designed
**PDGF**	NM_016205.3	F: GCCAGGTTGTCTCCTGGTTA R: TGCTTGGGACACATTGACAT	86 bp	40	Designed
**GAPDH**	NM_002046.7	F: TGCACCACCAACTGCTTAGC R: GGCATGGACTGTGGTCATGAG	87 bp	40	Designed
**SNORD47**	NR_003014.2	F: ACT GTA AAA CCG TTC CA	<100 bp	45	Designed
**hsa-miR-185-5p**	MIMAT0000455	F: AGAGAAAGGCAGTTCC	<100 bp	45	Designed

### Ethical consideration

The study proposal was approved by the ethics committee of Shahid Sadoughi university of Medical Sciences, Yazd, Iran (Code: IR.SSU.MEDICINE.REC.1395.300). Written informed consent was obtained from all participants.

### Statistical analysis

Statistical analysis was performed using GraphPad Prism 6 (GraphPad software, San Diego, California, USA). All the experiments were performed at least twice. Using the 2-ΔΔCT method, quantitative RT-PCR data were analyzed. Data were expressed as mean ± SEM and the results were scatter plots with a median value. For statistical comparison between the study groups, the two-tailed nonparametric Mann–Whitney post-hoc and Kruskal–Wallis tests was used. The receiver operating characteristic (ROC) curve was obtained and the area under the curve (AUC) was considered. Correlation analysis was performed using a two-tailed Spearman's rank correlation. A p-value < 0.05 was considered as statistically significant.

## 3. Results

Blood samples from 25 women with grade III-IV endometriosis (case group) and 25 women without endometriosis (control group) were screened for the expression of circulating miR-185-5p, *VEGF*, and *PDGF* target genes. The demographic characteristics of our study participants are presented in table II. As expected, the two groups had significant differences in the terms of body mass index (BMI) and cancer antigen 125 (p < 0.001). However, the groups did not show significant differences in human epididymis protein 4, carcinoembryonic antigen, white blood cells or hemoglobin (Table II).

**Figure 1:**
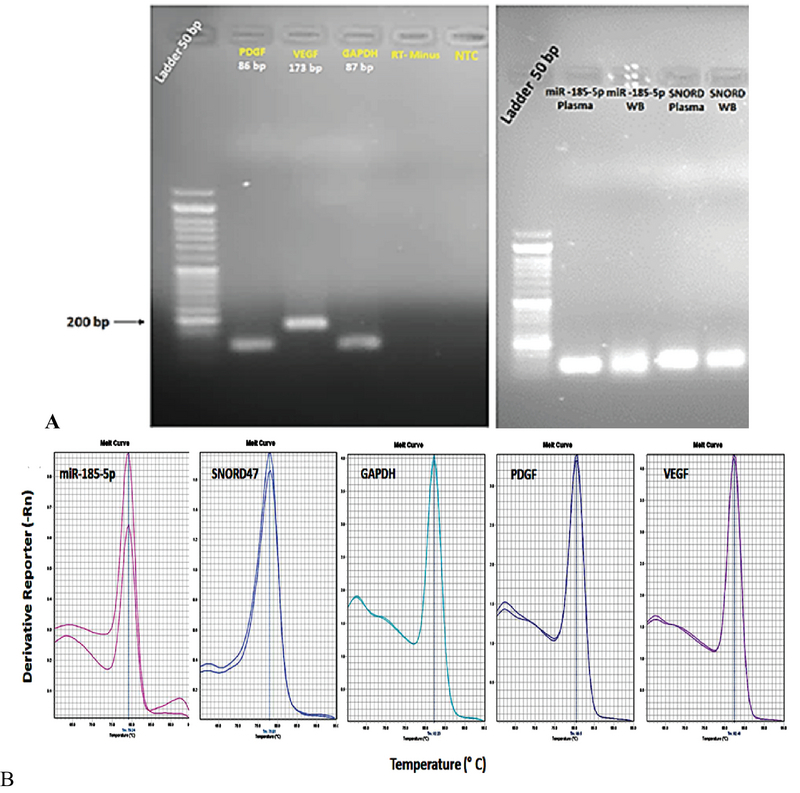
A) Agarose gel (1.5%) presenting the amplification of a specific PCR product of the expected size for each gene tested in this study. B) Specificity of real-time RT-PCR amplification after agarose gel electrophoresis. The melting curves of the miR-185-5p, PDGF, VEGF, and housekeeping genes including SNORD47 and GAPDH show a single pick.

For the evaluation of the quality (purity and intactness) and quantity (concentration and extraction efficiency) of extracted total RNA, the NanoDrop ND-1000 and 1.5% agarose gel electrophoresis were used (Figure1 a. The RNA purity was measured using a NanoDrop spectrophotometer, and the A260/A280 and A260/A230 ratios were used as indicators of different contaminants. Ratios between 1.8 and 2.0 for A260/A280 were accepted as indicating pure RNA (Table III). Contamination by DNA and protein was indicated by A260/A280 ratios above 2.0 or below 1.8. For the A260/A230 ratio, the acceptance range was between 1.8 and 2.2 (Table III).

**Figure 2:**
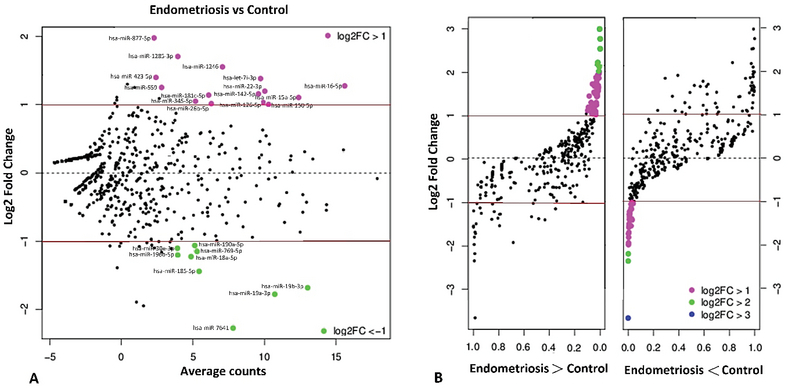
Volcano plot of miRNA analysis showing the aberrant expression of miRNAs in whole blood from women with and without endometriosis. A. Plots of log2 fold-change (FC) plot vs. average counts (AC) for differentially expressed miRNAs. The AC represents different miRNA expression between two study groups. The red lines indicate |log2 FC| = 1. B. Plots of log2 FC plot on *p* (significance level). The purple dots represent significant upregulated miRNA (|log2 FC| > 1, p < 0.015), the green dots represent significant downregulated miRNA (|log2 FC| > 2, p < 0.017), and the black dots represent no significance.

**Figure 3:**
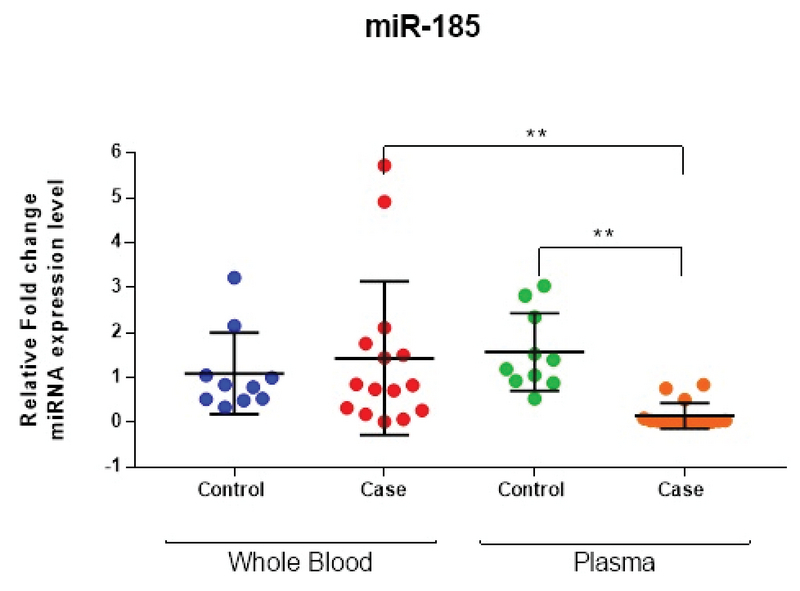
Quantitative real-time PCR analysis of miR-185-5p in whole blood and plasma in the case group compared to the controls (*n* = 25, pooled sample). The results are normalized relative to the expression level of the reference gene, SNORD47. Lines are drawn to demonstrate the median value, and the Mann-Whitney *U* test was used to calculate statistical significance (** p < 0.01, nonparametric Mann-Whitney test).

The specificity of the qRT-PCR products of the miR-185, target, and housekeeping genes was confirmed by the existence of a unique peak in the melting curve and the presence of a single band with a predictable size after agarose electrophoresis and SafeView nucleic acid staining (Figure 1 a, b). To evaluate the miRNA expression profiles in whole blood, total miRNAs were isolated, and miRNA expression profiling candidates were examined by Illumina-based deep sequencing. A total of 856 mature miRNAs were distinguished through small non-coding RNA sequencing analysis. The expression profiles of 30 miRNAs were found to be significantly different between the case and control groups (p < 0.016). Of these 30 miRNAs, 15 were upregulated and 15 were downregulated with endometriosis (Figure 2). From these miRNAs, the relative expression of miR-185-5p in the whole blood and plasma of endometriosis women is presented in Figure 3. On the other hand, the expression level of miR-185-5p in plasma was compared between the case and control groups (Figure 3).

### Plasma and whole blood expression levels of miR-185-5p

#### Comparison of miR-185-5p expression level in plasma and whole blood

**Figure 4:**
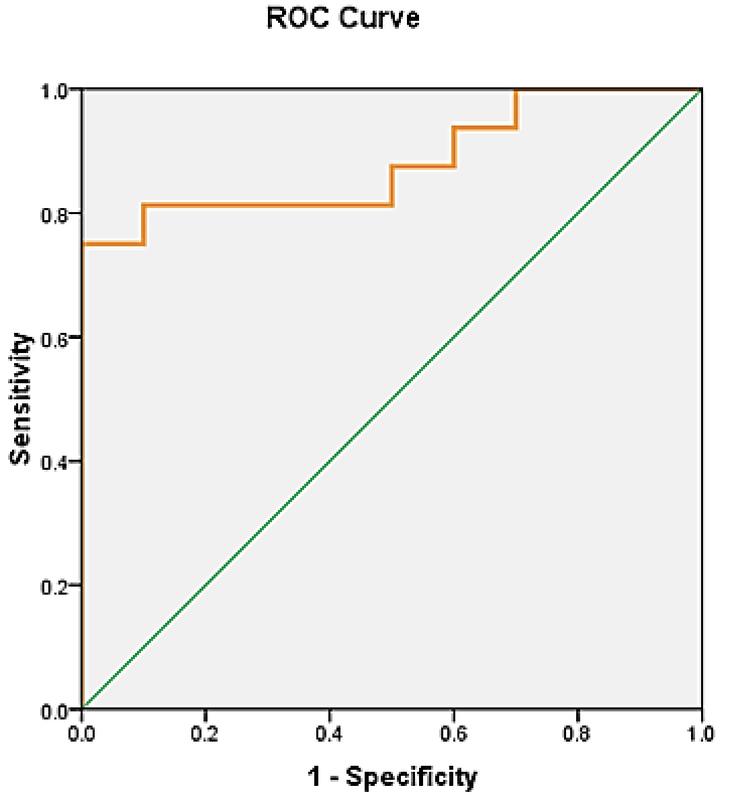
ROC curve analysis of miR-185 for endometriosis sample set analyzed for relative expression level of miR-185-5p in plasma. Best cut-off = 0.3750, sensitivity = 0.812, specificity = 0.900, 1-specifiity = 0.1, AUC = 0.919 (p < 0.001; 95% CI: 0.808-1.029).

The expression levels of miR-185-5p in plasma and whole blood were analyzed using qRT-PCR. The relative expression levels of candidate miRNA are shown in Figure 3. Compared with the sera of the control group, the expression of miR-185-5p in the serum was significantly lower in the case group (p < 0.001, RFC = 0.1469); however, no significant change was observed in miR-185-5p expression in whole blood from the cases compared with the control group (p = 0.73, RFC = 1.43). Conversely, plasma and whole blood levels of miR-185-5p were different, and the expression level of miR-185-5p in whole blood was significantly higher than in sera in the case group (p < 0.001) (Figure 3). An ROC curve was generated to analyze the capability of predicting endometriosis in women, and the AUC of miR-185-5p was 0.919 (95% confidence interval (CI): 0.808-1.029, specificity: 0.900, sensitivity: 0.812). The ROC curve analysis of miR-185-5p is shown in Figure 4.

### miR-185-5p as a potential biomarker of endometriosis

To determine the potency of miR-185-5p as a biomarker for endometriosis, an ROC curve was plotted. Remarkably, ROC analysis determined a cut-off value of 0.3750, with high values for sensitivity, specificity, and the AUC of 0.919 for miR-185-5p (P < 0.001; 95% CI: 0.808-1.029), proposing it as a potentially potent predictor for endometriosis (Figure 4).

### Comparison of PDGF and VEGF expression levels in whole blood

**Figure 5:**
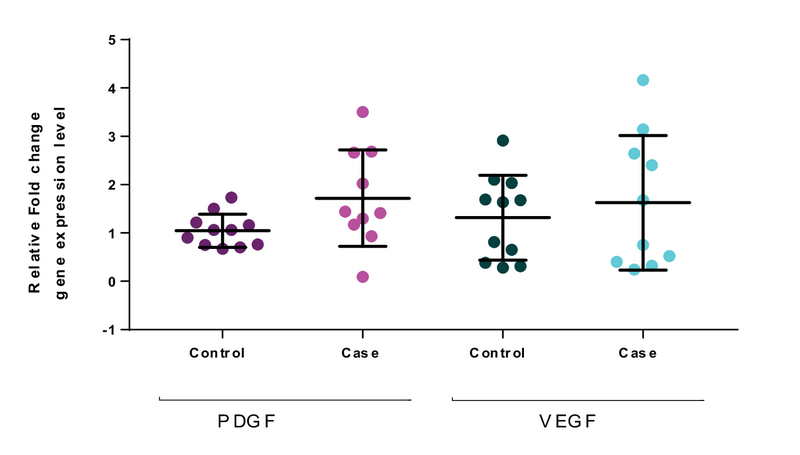
Quantitative real-time PCR analysis of PDGF and VEGF for whole blood of the case group (n = 25, pooled sample) compared to the controls (n = 25, pooled sample). The results are normalized relative to the expression level of the reference gene, GAPDH. Lines are drawn to demonstrate the median values, and the Mann-Whitney *U* test was used to calculate statistical significance.

The evaluation of the gene targets of *PDGF* and *VEGF* in whole blood showed no significant difference in mRNA levels in the case group compared with the controls. The results of RT-PCR demonstrated that compared with the controls, there was no significant decrease or increase in the expression of *VEGF* and *PDGF* mRNA in whole blood in the case group compared to the control group (*PDGF*, p = 0.0976 and *VEGF*, p = 0.3608). The expression of *VEGF-A* and *PDGF* is therefore not able to predict the clinical outcome (Figure 5).

**Table 2:** Baseline characteristics of the case and the control groups


**Characteristics**	**Case group (n=25)**	**Control group (n=25)**	P-value* **
**Age (years)**	5.8 ± 31.3	5.4 ± 29.3	0.19
**BMI (Kg/m${}^{2}$)**	3.1 ± 21.8	5 ± 25.2	<0.001
**CA125 (IU/ml) **	45.2 ± 54.4	11.2 ± 17.3	<0.001
**HE4 (pmol/l) **	12.3 ± 47.6	10 ± 44.7	0.36
**CEA (ng/ml) **	0.6 ± 1.2	0.7 ± 1.4	0.40
**WBC (count/μl) **	1735 ± 6634	1662 ± 6948	0.49
**Hb (g/dl) **	1.3 ± 12.20	1.2 ± 12.90	0.14
All data were presented as Mean ± SD, BMI: Body mass index; CA125: Cancer antigen 125; HE4: Human epididymis protein 4; CEA: Carcinoembryonic antigen; WBC: White blood cell; Hb: Hemoglobin, * Student's *t* test			

**Table 3:** Total RNA and miRNA quantity and purity control using the NanoDrop Spectrophotometer


**Sample**	**Concentration (ng/µl)**	**260/280 ratio**	**260/230 ratio**
**Total RNA from whole blood (control group)**	125.1	1.85	1.91
**Total miRNA from whole blood (case group)**	117.4	1.89	1.87

## 4. Discussion

The present study was undertaken to elucidate the expression level of miR-185-5p in the plasma and whole blood and its angiogenesis-related potential targets (*VEGF* and *PDGF*) in the whole blood of 25 women with endometriosis compared with the 25 women without endometriosis. A differential expression analysis of circulating miR-185-5p was conducted and predicted their downstream *VEGF* and *PDGF* target genes among the case and control groups using real-time quantitative RT-PCR to provide evidence of the value of miR-185-5p as a new diagnostic biomarker for endometriosis. The goal was to enhance the understanding of the gene expression pattern of miR-185-5p in plasma and whole blood and to assess the potency of this as a biomarker of disease progression in endometriosis.

The results showed that there was a lower expression of miR-185-5p in the plasma of the case group compared to the controls (Figure 3) and that there was no significant difference in the expression of *VEGF* and *PDGF* mRNA of whole blood in endometriosis patients compared to the control group (Figure 5). The results show that the mRNA expressions of *VEGF* and *PDGF* in whole blood were not statistically significant between the endometriosis and control groups (Figure 5), although increases of 3.12 *(PDGF)* and 2.33 *(VEGF)* in relative fold change were observed in the case groups.

To date, few studies have focused on the expression of *VEGF* and *PDGF* simultaneously in the whole blood of women suffering from endometriosis. Previous research findings relating to the gene expression pattern of *VEGF* and *PDGF* have been inconsistent and contradictory (19-22). The gene expression result of *VEGF* is similar to the results of Pellicer and colleagues who could not find a significant alternation in the mRNA expression of *interleukin-1β* and *VEGF* in women with endometriosis (23). Inconsistent results were identified regarding the gene expression and polymorphisms in the *VEGF* gene in the progression of endometriosis, which can be ascribed to methodological differences, sample size, qualified control type, using unadjusted risk estimates, and the heterogeneity of the people studied. In some studies, the serum level of the *VEGF* marker in endometriosis was found to be different from our recent results (24, 25). Some studies have shown an increase in the *VEGF* expression level and a significant difference in patients with endometriosis compared to the control group (26, 27). In another study, the *VEGF* level in different phases of the menstrual cycle (in patients who were assumed to have endometriosis) was compared with control groups, and no statistical difference was identified after the calculation of the phase of the cycle (28).

One of the advantages of our data was the simultaneous comparison of *VEGF* and *PDGF* expression in whole blood in women with endometriosis compared with the control group. Blood is a tissue and is made up of a variety of cells, each having a different job. In our study, we used whole blood for RNA extraction and miRNA profiling and gene expression quantification of target genes. The buffy coat compose less than 1% of the total volume of the blood, but most of the white blood cells and platelets aggregate in it and therefore it is a good source for RNA extraction. Plasma is considered as an acellular blood component; therefore, nucleic acids extracted from whole blood are mainly extracted from peripheral blood mononuclear cells, including T cells, B cells, NK cells and monocytes (29).

As previously mentioned, *PDGF* is a platelet-derived growth factor that is an effective mitogen and plays a critical role in the inflammatory response and in the proliferation and differentiation of progenitor cells. It has been confirmed that *PDGF* plays an essential role in several physiological processes such as wound healing, regeneration, cell proliferation, migration, and angiogenesis. It was reported that *PDGF* induced the differentiation of embryonic stem cells into endothelial cells (30) and it was indicated that *PDGF* is also involved in vascular development and tumor angiogenesis in pathological conditions (9, 31). In concert with other proangiogenic factors, *PDGF* induces the formation and spread of new vessels by recruitment of perivascular cells. It is said that overexpression of *PDGF* along with *VEGF* results in pericyte vessel formation and angiogenesis (32). It is also said that combined blockage of *VEGF, FGF* and *PDGF* cell signaling pathways suppresses angiogenesis in endometriosis lesions (33).

In our study, despite the expression of *PDGF* and *VEGF* being elevated in the whole blood of women with endometriosis, the increased mRNA levels of *PDGF* and *VEGF* were not statistically significant. This statistical insignificance could be the result of the small sample size; seemingly insignificant population differences might still find statistical significance. A larger and more varied population would lead to more meaningful results. Based on the above-mentioned reports, it might be anticipated that other miRNAs that target *PDGF* and *VEGF* and their receptors could describe molecular and cellular features of endometriotic lesions with a focus on the *VEGF*- and *PDGF*-signaling pathways.

According to the previous studies, it appears that angiogenic activity may be increased by the raised level of *VEGF* in the peritoneal fluid of endometriosis patients. These elevated levels of *VEGF* and *PDGF* possibly trigger proangiogenic mediator secretion and neovascularization within the peritoneal environment.

It seems that endometriosis is only related to pelvic inflammation and has nothing to do with the change of *VEGF* and *PDGF* levels in circulation. The current knowledge of specific miRNAs that regulate the *PDGF* and *VEGF* signal pathways favors the idea that this might be a remunerative target for the development of novel therapeutics. Considering that the subjects in this study had different types of clinical profile that could change the level of *VEGF* and *PDGF* in the blood in both the endometriosis and control groups, it can be said that the lack of significant difference observed in *VEGF* and *PDGF* could be due to these specific cases. Furthermore, the difference in these controversial results may be due to the small number of people involved in the study.

To avoid any difference in the results, fertile women free of gynecological diseases should be used to form an ideal control group. However, multi-centric studies with a suitable design involving different populations, together with the combined analysis of gene expression in *VEGF* and *PDGF*, are necessary to contribute to the understanding of this disease. Nonetheless, the results of the ROC analysis of miR-185-5p expression suggest a potentially non-invasive biomarker in the plasma of endometriosis patients that could reflect disease progression (Figure 4). Therefore, miR-185-5p could potentially serve as a diagnostic biomarker in the prognosis and diagnosis of endometriosis and for evaluation of therapeutic strategies for endometriosis at the molecular level. This will require further and larger-scale studies for confirmation.

## 5. Conclusion

Our findings proposed miR-185-5p as a novel biomarker and regulator in endometriosis pathogenesis, and these findings support the notion that target therapies against miR-185-5p should have priority in targeting *PDGF* and *VEGF*. They are therefore promising tools in therapeutic developments to control the progression and metastasis processes in endometriosis patients. Overall, the results of the statistical analysis suggest miR-185-5p as a potential biomarker of disease activity in endometriosis.

##  Conflict of Interest

The authors declare that there are no conflicts of interest.

## References

[1] Ya Panina, As Yakimov, Yk Komleva, Av Morgun, Ol Lopatina, Na Malinovskaya, .

[2] Zetter, Phd Bruce R. (1998). ANGIOGENESIS AND TUMOR METASTASIS. Annual Review of Medicine.

[3] Folkman J (2002). Role of angiogenesis in tumor growth and metastasis. Seminars in Oncology.

[4] Ribatti Domenico, Vacca Angelo, Presta Marco (2000). The discovery of angiogenic factors:. General Pharmacology: The Vascular System.

[5] Pugh Christopher W, Ratcliffe Peter J (2003). Regulation of angiogenesis by hypoxia: role of the HIF system. Nature Medicine.

[6] Ucuzian Areck A., Gassman Andrew A., East Andrea T., Greisler Howard P. (2010). Molecular Mediators of Angiogenesis. Journal of Burn Care & Research.

[7] Mustonen T. (1995). Endothelial receptor tyrosine kinases involved in angiogenesis. The Journal of Cell Biology.

[8] Sato Thomas N., Tozawa Yuzuru, Deutsch Urban, Wolburg-Buchholz Karen, Fujiwara Yuko, Gendron-Maguire Maureen, Gridley Thomas, Wolburg Hartwig, Risau Werner, Qin Ying (1995). Distinct roles of the receptor tyrosine kinases Tie-1 and Tie-2 in blood vessel formation. Nature.

[9] Raica Marius, Cimpean Anca Maria (2010). Platelet-Derived Growth Factor (PDGF)/PDGF Receptors (PDGFR) Axis as Target for Antitumor and Antiangiogenic Therapy. Pharmaceuticals.

[10] Lindblom P. (2003). Endothelial PDGF-B retention is required for proper investment of pericytes in the microvessel wall. Genes & Development.

[11] Armulik A. (2005). Endothelial/Pericyte Interactions. Circulation Research.

[12] M Presta, E Foglio, A Churruca Schuind, R. Ronca

[13] H Yoshiji, Harris, Up Thorgeirsson

[14] Guzick David S., Silliman Nancy Paul, Adamson G.David, Buttram Jr. Veasy C., Canis Michel, Malinak L.Russell, Schenken Robert S. (1997). Prediction of pregnancy in infertile women based on the American Society for Reproductive Medicine's revised classification of endometriosis. Fertility and Sterility.

[15] Pösel Claudia, Möller Karoline, Fröhlich Wenke, Schulz Isabell, Boltze Johannes, Wagner Daniel-Christoph (2012). Density Gradient Centrifugation Compromises Bone Marrow Mononuclear Cell Yield. PLoS ONE.

[16] Ling Guoyu, Waxman David J. (2013). Methods in Molecular Biology.

[17] Mcalexander Melissa A., Phillips Maggie J., Witwer Kenneth W. (2013). Comparison of Methods for miRNA Extraction from Plasma and Quantitative Recovery of RNA from Cerebrospinal Fluid. Frontiers in Genetics.

[18] Ra Alamro, M Mustafa, Ak Al-Asmari

[19] Agrawal Swati, Tapmeier Thomas, Rahmioglu Nilufer, Kirtley Shona, Zondervan Krina, Becker Christian (2018). The miRNA Mirage: How Close Are We to Finding a Non-Invasive Diagnostic Biomarker in Endometriosis? A Systematic Review. International Journal of Molecular Sciences.

[20] Laschke Matthias W, Menger Michael D (2018). Basic mechanisms of vascularization in endometriosis and their clinical implications. Human Reproduction Update.

[21] Gagne D. (2003). Levels of vascular endothelial growth factor (VEGF) in serum of patients with endometriosis. Human Reproduction.

[22] Xj Liu, Xg Bai, Yl Teng, L Song, N Lu, Rq Yang

[23] Pellicer Antonio, Albert Carmela, Mercader Amparo, Bonilla-Musoles Fernando, Remohí José, Simón Carlos (1998). The follicular and endocrine environment in women with endometriosis: local and systemic cytokine production. Fertility and Sterility.

[24] Acimovic Milena, Vidakovic Snezana, Milic Natasa, Jeremic Katarina, Markovic Milos, Milosevic-Djeric Ana, Lazovic-Radonjic Gordana (2016). Survivin and Vegf as Novel Biomarkers in Diagnosis of Endometriosis/ Survivin i vegf kao novi biomarkeri u dijagnostici endometrioze. Journal of Medical Biochemistry.

[25] Baranov Vladislav S., Ivaschenko Tatyana E., Liehr Thomas, Yarmolinskaya Maria I. (2015). Systems genetics view of endometriosis: a common complex disorder. European Journal of Obstetrics & Gynecology and Reproductive Biology.

[26] Ferrero Simone (2004). Matalliotakis IM, Goumenou AG, Koumantakis GE, Neonaki MA, Koumantakis EE, Dionyssopoulou E, Athanassakis I, Vassiliadis S. Serum concentrations of growth factors in women with and without endometriosis: the action of anti-endometriosis medicines [Int Immunopharmacol 2003; 3(1):81–89]. International Immunopharmacology.

[27] Mclaren J., Prentice A., Charnock-Jones D.S., Smith S.K. (1996). Vascular endothelial growth factor (VEGF) concentrations are elevated in peritoneal fluid of women with endometriosis. Human Reproduction.

[28] Pupo-Nogueira A., De Oliveira R.M., Petta C.A., Podgaec S., Dias J.A., Abrao M.S. (2007). Vascular endothelial growth factor concentrations in the serum and peritoneal fluid of women with endometriosis. International Journal of Gynecology & Obstetrics.

[29] Malhotra Neena, Karmakar Debjyoti, Tripathi Vishwas, Luthra Kalpana, Kumar Sunesh (2011). Correlation of angiogenic cytokines-leptin and IL-8 in stage, type and presentation of endometriosis. Gynecological Endocrinology.

[30] Lange S., Heger J., Euler G., Wartenberg M., Piper H. M., Sauer H. (2008). Platelet-derived growth factor BB stimulates vasculogenesis of embryonic stem cell-derived endothelial cells by calcium-mediated generation of reactive oxygen species. Cardiovascular Research.

[31] Cao Renhai, Bråkenhielm Ebba, Li Xuri, Pietras Kristian, Widenfalk Johan, Östman Arne, Eriksson Ulf, Cao Yihai (2002). Angiogenesis stimulated by PDGF-CC, a novel member in the PDGF family, involves activation of PDGFR-αα and -αβ receptors. The FASEB Journal.

[32] Magnusson P. U., Looman C., Ahgren A., Wu Y., Claesson-Welsh L., Heuchel R. L. (2007). Platelet-Derived Growth Factor Receptor-  Constitutive Activity Promotes Angiogenesis In Vivo and In Vitro. Arteriosclerosis, Thrombosis, and Vascular Biology.

[33] Laschke M.W., Elitzsch A., Vollmar B., Vajkoczy P., Menger M.D. (2005). Combined inhibition of vascular endothelial growth factor (VEGF), fibroblast growth factor and platelet-derived growth factor, but not inhibition of VEGF alone, effectively suppresses angiogenesis and vessel maturation in endometriotic lesions. Human Reproduction.

